# Second and third delays in emergency obstetric care: predictors in Northwest Cameroon

**DOI:** 10.1186/s12884-026-08962-0

**Published:** 2026-05-06

**Authors:** Tosi Jones Nkwain, Lifoter Kenneth Navti, Mary Bi Suh Atanga

**Affiliations:** 1 Department of Public Health, Faculty of Health Sciences, The University of Bamenda, Bamenda, Cameroon; 2 Department of Biochemistry, Faculty of Science, The University of Bamenda, Bamenda, Cameroon; 3 Department of Nursing, Faculty of Health Sciences, The University of Bamenda, Bamenda, Cameroon

**Keywords:** Emergency obstetric care, Three delays model, Conflict-affected region, Socioeconomic barriers, Cameroon

## Abstract

**Background:**

Maternal mortality in Cameroon’s conflict-affected Northwest Region remains high, with delays in accessing and receiving emergency obstetric care (EmOC) being a major contributor. Using the Three Delays Model, this study investigated socioeconomic and health system predictors of the second delay (reaching a health facility after deciding to seek care) and third delay (receiving adequate care upon arrival) among postnatal women in referral hospitals.

**Methods:**

A hospital-based cross-sectional study was conducted from June 2024 to April 2025 (11 months) among 472 postnatal women across eight high-volume public referral facilities in the Northwest Region. Data were collected using a structured, interviewer-administered questionnaire adapted from validated tools. Delays were defined using a two-stage approach: an initial screening question followed by time thresholds (> 1 h from decision to arrival for the second delay; >1 h from arrival to care for the third delay), with additional contributing barriers assessed via closed-ended yes/no questions. Univariable chi-square analysis and multivariable binary logistic regression were performed in SPSS version 25 to identify independent predictors. Statistical significance was set at *p* < 0.05.

**Results:**

Second delay affected 124 women (26.3%) and was independently predicted by rural residence (AOR = 2.82, 95% CI: 1.79–4.45, *p* < 0.001), semi-urban residence (AOR = 2.04, 95% CI: 1.23–3.38, *p* = 0.005), and low household income (< 25,000 FCFA) (AOR = 3.46, 95% CI: 1.66–7.21, *p* = 0.001). Third delay affected 154 women (32.6%), with lower odds among women aged 20–29 years (AOR = 0.42, 95% CI: 0.22–0.80, *p* = 0.008), those aged 30–39 years (AOR = 0.51, 95% CI: 0.28–0.93, *p* = 0.029), married women (AOR = 0.42, 95% CI: 0.26–0.68, *p* < 0.001), and women earning 51,000–100,000 FCFA (AOR = 0.39, 95% CI: 0.19–0.80, *p* = 0.010). Self-reported barriers included staffing shortages (40.2%), poor service quality (50.8%), blood supply issues (33.9%), and long admission processes (33.7%).

**Conclusion:**

Over one-quarter of women experienced delays in reaching EmOC facilities, driven by rural residence and low income, while one-third faced delays in receiving care, associated with lower odds among married women and those with moderate income. Self-reported systemic barriers, including staffing shortages, poor infrastructure, and inefficient referrals, compound socioeconomic vulnerabilities in this conflict-affected zone. Targeted interventions, such as subsidised rural transport, digital referral systems, and enhanced facility readiness, are urgently needed to reduce preventable maternal deaths and advance Cameroon’s SDG 3.1 commitments.

**Supplementary Information:**

The online version contains supplementary material available at 10.1186/s12884-026-08962-0.

## Introduction

 Maternal mortality is defined as the death of a woman while pregnant or within 42 days of termination of pregnancy, irrespective of the duration and site of the pregnancy, from any cause related to or aggravated by the pregnancy or its management (but not from accidental or incidental causes) [1]. Maternal mortality remains a pressing global health challenge, with an estimated 260,000 women dying from pregnancy-related causes in 2023, the majority in sub-Saharan Africa [1].

Most deaths stem from preventable obstetric complications such as haemorrhage, eclampsia, sepsis, obstructed labour, and unsafe abortion, where timely access to emergency obstetric care (EmOC) is critical [2]. EmOC encompasses basic interventions (e.g., uterotonics, neonatal resuscitation) and comprehensive services (e.g., cesarean sections, blood transfusions), yet coverage in low- and middle-income countries (LMICs) is limited by systemic and socioeconomic barriers [2, 3]. In conflict-affected regions, these challenges are worsened by insecurity, displacement, and disrupted health systems, disproportionately affecting marginalised women.

The Three Delays Model provides a framework to understand barriers to EmOC: (1) delay in deciding to seek care, (2) delay in reaching a health facility, (3) delay in receiving adequate care upon arrival [4].

The second delay involves structural barriers, long distances, poor transport infrastructure, unaffordable costs, and insecurity, while the third delay arises from facility-level issues like staff shortages, equipment failures, stock-outs, and inefficient referrals [5, 6]. Socioeconomic factors, such as low income, limited education, and unemployment, worsen delays by restricting financial resources and decision-making autonomy [7, 8]. Rural women face heightened risks due to geographic isolation and limited health infrastructure, often compounded by cultural norms and distrust in facility care quality [8, 9]. In conflict settings, attacks on health facilities and restricted mobility further delay access to and delivery of care [10].

In Cameroon, the maternal mortality ratio of 258 per 100,000 live births in 2023 reflects persistent inequities, with rural and conflict-affected regions far from the SDG 3.1 target of < 140 by 2030 [1, 11]. Global analyses confirm that socioeconomic and health system determinants continue to drive transitions in maternal mortality, with persistent gaps in LMICs [12]. The Northwest Region, engulfed by the Anglophone crisis since 2016, faces unique challenges: damaged roads, curfews, and health worker shortages disrupt transport and service delivery [13, 14]. Studies in sub-Saharan Africa have explored general maternal health access. Still, few focus on the second and third delays in Cameroon’s conflict zones, where socioeconomic (income, education, occupation) and health system factors (staffing, referrals, service quality) interact uniquely [2, 15]. Similar evaluations of EmONC services in other Cameroonian regions have highlighted persistent gaps in availability and quality [13]. For example, a Nigerian study found rural women faced triple the odds of transport delays, while facility delays were linked to stock-outs in 40% of cases [2]. In Cameroon, evidence remains scarce, hindering targeted interventions.

This study aims to identify socioeconomic (income, education, occupation, urbanisation) and health system (service availability, staff capacity, referrals) predictors of delays in reaching and receiving EmOC among postnatal women in eight referral hospitals in Northwest Cameroon. By generating context-specific evidence, we seek to inform policies and interventions to reduce maternal mortality in fragile settings, aligning with Cameroon’s SDG commitments [16]. The main objective of the study was to identify factors contributing to the second and third delay.

## Materials and methods

### Study design

A hospital-based cross-sectional study was conducted from June 2024 to April 2025 (11 months) among 472 postnatal women across eight high-volume public referral facilities in the Northwest Region. This period was selected to account for seasonal challenges (rainy-season impacts on access) and security dynamics in the Northwest Region, ensuring feasible and safe data collection while capturing representative EmOC experiences across referral facilities.

### Study area

The study was conducted in the Northwest Region of Cameroon, one of the two English-speaking regions of the country. The Northwest Region is located in the western highlands of Cameroon (approximate coordinates: 6°20′N, 10°30′E), predominantly rural with challenging terrain and road networks. Health services are organised in three levels: primary health care at health centres, secondary care at district hospitals, and tertiary care at regional hospitals.

For this study, eight public health facilities were purposively selected based on their high delivery volumes in 2023 and their role as major referral centres for emergency obstetric care in the region (unpublished statistics from the regional delegation of Public Health). These facilities included Bamenda Regional Hospital, Nkwen District Hospital, Tubah District Hospital, Ndop District Hospital, Santa District Hospital, Nkambe Regional Hospital Annexe, Ndu District Hospital, and Fundong District Hospital. Together, they serve a wide catchment area that covers both urban and rural populations across multiple health districts, manage the majority of complicated deliveries, and receive referrals from peripheral health centres. By including these strategically located and high-volume facilities, the study was able to capture the experiences and systemic factors influencing delays in emergency obstetric care that are a reflection of patterns across the Northwest Region.

### Study population

Postnatal women who had given birth at Bamenda Regional Hospital, Nkwen District Hospital, Tubah District Hospital, Ndop District Hospital, Santa District Hospital, Nkambe Regional Hospital Annexe, Ndu District Hospital and Fundong District Hospital during the data collection period.

### Eligibility criteria

All postnatal women who had given birth in the selected hospitals during the data collection period, and who provided verbal informed consent after explanation, were included in the study. Postnatal women who were critically ill or unconscious were excluded. During analysis, the data were cleaned, and records with incomplete information were removed.

### Sample size and sampling technique

#### Sample size calculation

The sample size was calculated using G*Power version 3.1.9.6 (Heinrich Heine Universität Düsseldorf, Germany) for logistic regression with the dependent variable ‘delay in reaching the health facility’ (second delay, yes/no). Assuming an odds ratio (OR) of 1.5 for rural-urban disparities, based on prior sub-Saharan African studies [2], with 80% power (1–β = 0.80) and a two-sided α = 0.05, the minimum required sample was 360. To account for an anticipated 80% response rate, the minimum sample size was adjusted to 450. The final sample size achieved was 472, with a 100% completion rate among eligible women who consented.

#### Sampling technique

A multistage sampling technique was used. Firstly, 6 District Hospitals and two Regional Hospitals were purposively selected. Secondly, to derive the final sample, the calculated sample size was proportionally allocated to the selected hospitals based on the number of deliveries. At the third stage, postnatal women were conveniently sampled in the different Hospitals.

#### Data collection

Data were collected using a structured, interviewer-administered questionnaire adapted from validated tools [2]. The tool covered socio-demographic information and factors influencing emergency obstetric care decision-making. Data collection was conducted using Kobo Collect, which allowed for electronic data entry and real-time monitoring to enhance accuracy and completeness.

#### Validity and reliability

Pre-testing was conducted at Ako District Hospital with 20 postnatal women to ensure questionnaire reliability and clarity, and corrections were made. Additionally, supervisors reviewed and validated the questionnaire. The questionnaire was adapted from validated tools used in similar LMIC studies [2].

#### Data collection procedure

Data were collected using a structured questionnaire adapted to the local context and informed by relevant literature. A total of 20 nurses and midwives from different health facilities were trained for one day on the study procedures and the use of Kobo Collect for electronic data collection. Following training, the data collectors interviewed postnatal women after obtaining verbal consent, with a clear explanation of the study procedures. Each interview lasted approximately 10–15 min. Interviews were conducted in English or Pidgin English, depending on the respondent’s preferred language. The principal investigator supervised the entire process to ensure consistency, accuracy, and adherence to study protocols.

### Variable description

#### Dependent variable

Delays were assessed using a two-stage approach adapted from validated tools employed in sub-Saharan Africa [17]. First, participants were asked a screening question: “After deciding to seek emergency obstetric care, have you ever experienced a delay in reaching a health facility or in receiving care at the facility?” (yes/no response). Women who answered “yes” proceeded to the second stage for further evaluation.

Second delay (delay in reaching the health facility after deciding to seek care) was classified as present if the reported time from decision to arrival exceeded 1 h (> 1 h), reflecting significant access barriers. This time threshold was adapted from Wanaka et al. (2020) [17] and similar studies in rural sub-Saharan settings. Additional contributing aspects were assessed using closed-ended yes/no questions, including lack of available or affordable transport, unreliable/public transport, poor road conditions, insecurity or conflict-related risks, unsafe or difficult travel at night, and ghost town/lockdown operations (enforced closures restricting movement). These are detailed in Table 2.

The third delay (delay in receiving adequate care upon arrival at the facility) was classified as present if the reported time from arrival to receiving care exceeded 1 h (> 1 h), indicating substantial facility-level constraints. This threshold was also adapted from Wanaka et al. (2020) [17]. Additional contributing barriers were assessed through closed-ended yes/no questions, including shortage of health staff or long waiting time for a provider, inadequate or poor-quality service delivery, lack of essential equipment or supplies, blood stock-outs or unavailability of transfusion services, long admission or registration process, delay in referral or transfer within or between facilities, absence of necessary medications (e.g., uterotonics or antihypertensives), poor infrastructure (e.g., no electricity, water, or functional operating theatre), informal payments or demands for bribes, and perceived poor attitude or discrimination by staff. Additional contributing barriers (e.g., staff shortages, poor infrastructure, blood supply issues) were self-reported by women respondents via closed-ended yes/no questions and were not extracted from facility records or independently verified by health workers. These are presented in Table 5.

#### Independent variables

Age, level of education, occupation, religion, level of urbanisation, marital status, monthly household income.

### Data processing and analysis

Data collectors submitted completed forms to the central server, where datasets were downloaded and stored in Microsoft Excel. The data were cleaned and coded, then exported to SPSS version 25.0 for statistical analysis. Descriptive statistics were used to summarise categorical variables using frequencies and percentages. Associations between explanatory factors and delays were examined using chi-square tests at the univariable level. Variables with a p-value < 0.05 in univariable analysis were entered into multivariable binary logistic regression models to estimate adjusted odds ratios (ORs) and 95% confidence intervals (CIs). Statistical significance was set at *p* < 0.05.

### Ethical considerations

Ethical approval for this study was obtained from the Institutional Review Board of the Faculty of Health Sciences, University of Bamenda (Ref: 2022/0514H/UBa/IRB), and administrative authorisation was granted by the Regional Delegation of Public Health for the North West Region of Cameroon. Confidentiality was maintained by using codes instead of names, and participation was voluntary.

Verbal informed consent was obtained due to potential literacy challenges and cultural sensitivities in the study population. Consent processes were explained in pidgin or English, documented by the trained data collector, and overseen by the principal investigator to ensure comprehension and voluntariness.

## Results

### Sociodemographic characteristics of the study participants

A total of 472 postnatal women participated in the study. The mean age was 29 years (SD = 6.54), with the majority aged 20–29 years (45.6%) and 30–39 years (40.2%). Residence was urban for 46.4%, rural for 30.7%, and semi-urban for 22.9%. Most had secondary education (51.9%), were self-employed (44.9%), Christian (74.5%), and married (66.7%). Household monthly income was < 25,000 FCFA for 40.3% (Table 1).

**Table 1:** The sociodemographic characteristics of the study participants

Variable	Category	Frequency (*N* = 472)	Percentage (%)
Age (years)	< 20	30	6.4
	20–29 years	215	45.6
	30–39	190	40.2
	40–49	37	7.8
Level of Urbanization	Rural	145	30.7
	Semi urban	108	22.9
	Urban	219	46.4
Level of education	No formal education	15	3.1
	Primary	75	15.9
	Secondary	245	51.9
Occupation	Civil servant	63	13.3
	Housewife	102	21.6
	Private entity worker	25	5.3
	Self employed	212	44.9
	Student	70	14.8
Religion	Christian	352	74.5
	Muslim	120	25.4
Marital status	Married	315	66.7
	Single	149	31.8
	Widowed	7	1.5
Monthly household income (FCFA)	Less than 25,000	190	40.3
	25,000–50,000	133	28.2
	51000-100,000	85	18.0
	More than 100,000	64	13.6

### Second delay (delay in reaching the health facility)

Overall, 124 women (26.3%) experienced a second delay. After adjustment in multivariable binary logistic regression, independent predictors of second delay included rural residence (AOR = 2.82, 95% CI: 1.79–4.45, *p* < 0.001), semi-urban residence (AOR = 2.04, 95% CI: 1.23–3.38, *p* = 0.005), low household income (< 25,000 FCFA: AOR = 3.46, 95% CI: 1.66–7.21, *p* = 0.001; 25,000–50,000 FCFA: AOR = 2.46, 95% CI: 1.23–4.92, *p* = 0.020), no formal/primary education (AOR = 2.14, 95% CI: 1.23–3.73, *p* = 0.017), housewife occupation (AOR = 1.85, 95% CI: 1.02–3.36, *p* = 0.043), and private sector employment (AOR = 1.97, 95% CI: 1.04–3.70, *p* = 0.040) (Table 2). Self-reported contributing barriers to the second delay are presented in Table 3. Univariable associations are presented in Supplementary Tables S1 and S2.

**Table 2:** Multivariable analysis identifying predictors of delay in reaching an appropriate health facility

Variable	Category	AOR	95% CI	*p*-value
Monthly household income (FCFA)	< 25,000	3.46	1.66–7.21	**< 0.001**
	25,000–50,000	2.46	1.23–4.92	**0.020**
	51,000–100,000	1.93	0.87–4.30	0.108
	> 100,000	Ref	–	–
Level of urbanisation	Rural	2.82	1.79–4.45	**0.001**
	Semi-urban	2.04	1.23–3.38	**0.005**
	Urban	Ref	–	–
Education	No formal/Primary	2.14	1.23–3.73	**0.017**
	Secondary	0.93	0.58–1.48	0.780
	Tertiary	Ref	–	–
Occupation	Housewife	1.85	1.02–3.36	**0.043**
	Self-employed	1.36	0.53–3.50	0.520
	Private employed	1.97	1.04–3.70	**0.040**
	Student	0.83	0.36–1.92	0.532
	Government employed	Ref	–	–

*AOR* Adjusted Odd Ratios, *CI *Confidence Interval

**Table 3:** Self-reported contributing barriers to the second delay (*N* = 472)

Barrier Description	Yes *n* (%)	No *n* (%)
Unavailability of transport	161 (34.1)	311 (65.9)
Unreliable or unaffordable public transport	309 (65.6)	163 (34.4)
Poor road conditions	273 (57.8)	199 (42.2)
Insecurity or conflict-related risks	291 (61.7)	181 (38.3)
Unsafe or difficult travel at night	291 (61.7)	181 (38.3)
Ghost town/lockdown restrictions	100 (21.2)	372 (78.8)

### Third delay (delay in receiving adequate care at the health facility)

Overall, 154 women (32.6%) experienced a third delay. In the adjusted multivariable model, lower odds of third delay were independently associated with age 20–29 years (AOR = 0.42, 95% CI: 0.22–0.80, *p* = 0.008), age 30–39 years (AOR = 0.51, 95% CI: 0.28–0.93, *p* = 0.029), being married (AOR = 0.42, 95% CI: 0.26–0.68, *p* < 0.001), and monthly income of 51,000–100,000 FCFA (AOR = 0.39, 95% CI: 0.19–0.80, *p* = 0.010). Occupation was generally not a strong predictor after adjustment, although housewives had higher odds of experiencing a third delay (AOR = 1.92, 95% CI: 1.06–3.48, *p* = 0.032) compared with government-employed women (Table 4). Self-reported contributing barriers to the third delay are presented in Table 5. Univariable associations are presented in Supplementary Tables S1 and S2.

**Table 4:** Multivariable analysis identifying predictors of delay in receiving adequate care at the health facility

Variable	Category	AOR	95% CI	*p*-value
Age (years)	< 20	0.68	0.31–1.49	0.336
	20–29	**0.42**	**0.22–0.80**	**0.008**
	30–39	**0.51**	**0.28–0.93**	**0.029**
	> 40	Ref	–	–
Marital status	Married	**0.42**	**0.26–0.68**	**0.001**
	Widowed	0.88	0.21–3.66	0.860
	Single	Ref	–	–
Occupation	Housewife	**1.92**	**1.06–3.48**	**0.032**
	Student	1.45	0.78–2.69	0.241
	Self-employed	1.12	0.68–1.85	0.651
	Private employed	1.38	0.62–3.08	0.428
	Government employed	Ref	–	–
Monthly household income (FCFA)	< 25,000	1.44	0.78–2.66	0.245
	25,000–50,000	1.61	0.89–2.91	0.115
	51,000–100,000	**0.39**	**0.19–0.80**	**0.010**
	> 100,000	Ref	–	–

*AOR* Adjusted Odd Ratios, *CI *Confidence Interval

**Table 5:** Self-reported contributing barriers to the third delay (*N* = 472)

Barrier Description	Yes *n* (%)	No *n* (%)
Poor quality of services	240 (50.8)	232 (49.2)
Limited availability of services (e.g., equipment, supplies)	169 (35.8)	302 (64.0)
Shortage of staff	190 (40.2)	282 (59.8)
High workload contributing to delays	226 (47.9)	246 (52.1)
Delay in initiating treatment	168 (35.6)	304 (64.4)
Late or multiple referrals	141 (29.9)	331 (70.1)
Delay in receiving treatment	163 (34.5)	307 (65.0)
Difficulty obtaining blood supply	160 (33.9)	311 (66.1)
Long admission process	159 (33.7)	312 (66.3)
Perceived poor attitude or discrimination by staff	113 (23.9)	358 (76.1)

## Discussion

This study examined socioeconomic and health system predictors of the second and third delays in accessing and receiving emergency obstetric care (EmOC) among postnatal women in Northwest Cameroon, a region marked by the ongoing Anglophone crisis. Using a two-stage approach (initial screening question followed by time thresholds: >1 h from decision to arrival for second delay, > 1 h from arrival to care for third delay), with additional contributing barriers assessed through closed-ended questions, we found that 26.3% of women experienced delays in reaching facilities, driven primarily by rural residence and low income, while 32.6% faced delays in receiving care upon arrival, with younger age, marriage, and moderate income associated with lower odds of delay. These findings highlight the interplay of structural inequities and systemic weaknesses in a conflict-affected setting. These findings indicate that delays in emergency obstetric care in Northwest Cameroon are not solely clinical challenges but reflect broader structural inequities in transport systems, household economic capacity, and facility readiness, underscoring the need for integrated health system strengthening to reduce preventable maternal deaths.

The second delay prevalence (26.3%) is consistent with Wanaka et al. [17], who reported 31.7% in rural southern Ethiopia using similar composite barrier scoring, underscoring rural transport and income barriers in sub-Saharan contexts. This was lower than in some sub-Saharan African studies, where 40–50% of women face access barriers due to geographic and financial constraints [5, 18]. Rural residence (AOR = 2.82, 95% CI: 1.79–4.45, *p* < 0.001) and household income below 25,000 FCFA (AOR = 3.46, 95% CI: 1.66–7.21, *p* = 0.001) were the strongest predictors, consistent with evidence from Nigeria where rural women faced triple the odds of transport delays due to poor infrastructure and insecurity [2]. The composite score, capturing barriers such as lack of transport, unaffordable costs, long distances, poor roads, conflict-related insecurity, and unsafe night travel (Table 3), reflects the compounded challenges in Northwest Cameroon, where the crisis disrupts mobility and amplifies fear [14]. A South Sudan study similarly found that conflict reduced ambulance access and heightened perceived risks [19]. The significant role of low income aligns with findings from Burkina Faso, where cost barriers delayed 30% of rural women (9). The composite barrier approach, validated in Timor-Leste and Nigeria [2, 7], provides a robust, context-sensitive measure beyond simple time thresholds, which are prone to recall bias in retrospective surveys [6].

For the third delay, lower odds were observed among women aged 20–29 years (AOR = 0.42, 95% CI: 0.22–0.80, *p* = 0.008), married women (AOR = 0.42, 95% CI: 0.26–0.68, *p* < 0.001), and those earning 51,000–100,000 FCFA (AOR = 0.39, 95% CI: 0.19–0.80, *p* = 0.010). Marriage may reduce the odds of delay through spousal support in navigating facility bottlenecks, as observed in Ghana, where partners facilitated faster care [8]. Younger women may experience lower odds due to perceived clinical urgency or greater assertiveness in demanding care, though this requires further exploration [7]. The income effect suggests that moderate earners may avoid informal payment delays that burden the poorest, consistent with Ethiopian studies linking financial constraints to prolonged waiting times [18]. High self-reported prevalence of barriers such as staff shortages (40.2%), poor service quality (50.8%), blood supply issues (33.9%), and long admission processes (33.7%) (Table 5) mirrors systemic strain seen in Uganda, where 35% of facilities lacked essential EmOC supplies [20]. Conflict-related staff burnout and resource depletion likely exacerbate these inefficiencies [19].

The Three Delays Model [4] remains a vital framework for maternal mortality reduction. Our two-stage composite method effectively captures cumulative socioeconomic and systemic barriers, particularly in fragile settings where insecurity and distrust amplify delays [14]. For second delays, subsidised rural transport and community ambulance systems, successful in reducing access delays by 20% in Mozambique [21] are critical. For the third delay, facility readiness must be strengthened through staff training, reliable blood banks, and streamlined referrals, as demonstrated in Rwanda [3]. In Northwest Cameroon, integrating digital referral platforms with peace-building initiatives could mitigate conflict-related disruptions. Community engagement, including male involvement, is essential to address household-level barriers [8]. Policy efforts must prioritise rural infrastructure, financial protection, and conflict-resilient health systems to align with Cameroon’s SDG 3.1 commitments and reduce preventable maternal deaths [1].

### Recommendations

To reduce second delays associated with rural residence and low income (key predictors in the results), expand and subsidise ambulance services while establishing community-based transport networks, particularly for women in rural communities. To address third delays driven by reported facility barriers (e.g., staffing shortages, poor service quality, and weak referrals), increase staffing levels, provide targeted training in Emergency Obstetric and Newborn Care (EmONC) protocols to improve timely care, ensure consistent supplies of blood and functional equipment, and develop real-time digital communication channels and standardised referral protocols between facilities to facilitate streamlined processes in this conflict-affected setting.

### Limitations

This study has several limitations. First, the cross-sectional design limits the ability to draw causal inferences between predictors such as income or rural residence and the observed delays, highlighting the need for longitudinal research to confirm these associations. Second, the assessment of delays relied on self-reported data obtained through screening questions and composite scores, which may be subject to recall bias, particularly when women report retrospective travel or waiting times. Third, the hospital-based sampling strategy may have excluded women who delivered at home or in peripheral facilities, potentially underestimating delays experienced by more marginalised populations. In addition, facility-related barriers (e.g., staffing shortages, equipment availability, and service quality) were based solely on women’s self-reports rather than facility records or health worker confirmation, which may introduce reporting bias. Seasonality effects, such as poor road accessibility during the rainy season, may also not have been fully captured since data collection spanned 11 months rather than a complete calendar year. Finally, although the composite barrier score used in this study has been validated in similar contexts, it relies on perceived barriers and may not fully capture objective time-to-care measurements.

## Conclusion

This study reveals that second and third delays in accessing emergency obstetric care remain prevalent in Northwest Cameroon. The second delay was mainly associated with low household income and rural residence, reflecting barriers such as long travel times and poor transportation in the composite score. In contrast, the third delay was linked to facility-level issues, including inadequate staffing, limited equipment and supplies, and weak referral processes, as reported in the barriers (Tables 3 and 5). Strengthening referral networks, improving facility readiness, and integrating digital tools for real-time communication between facilities can help minimise delays. Addressing these gaps is critical for reducing preventable maternal deaths and advancing Cameroon’s progress toward SDG 3.1 on maternal health.

### Suggestion for further studies

Future research should adopt community-based approaches to explore women’s and families’ perceptions of emergency obstetric care and the barriers influencing timely decision-making. Studies assessing health system readiness, including staffing levels, equipment availability, blood supply, and ambulance services, across different levels of care would provide deeper insights into facility-level capacity to manage obstetric emergencies. Further investigation into the efficiency of referral systems between lower-level and higher-level facilities is also warranted. Comparative analyses between rural and urban health facilities could help elucidate contextual differences that shape the causes and patterns of delays in emergency obstetric care.

## Supplementary Information

Supplementary Material 1.

## Data Availability

The data supporting the findings of this study are available from the corresponding author (Tosi Jones Nkwain, tosijones94@gmail.com) upon reasonable request. Access is subject to approval by the Institutional Review Board of the Faculty of Health Sciences, The University of Bamenda, and relevant hospital administrations to ensure participant confidentiality and compliance with ethical guidelines.
