# Supplementary material for: Second and third delays in emergency obstetric care: predictors in Northwest Cameroon
Source: BMC Pregnancy Childbirth. 2026 May 6;26:495. doi: 10.1186/s12884-026-08962-0 (PMC13151339; doi:10.1186/s12884-026-08962-0)
Supplement: Supplementary file 1 — Supplementary Material 1. [file 12884_2026_8962_MOESM1_ESM.pdf]

Supplementary Table S1: Univariable analysis of factors associated with the second delay (delay in reaching an appropriate health facility)

Univariable chi-square associations between sociodemographic variables and the second delay (N=472). Variables with $p < 0.05$ were considered for multivariable modelling.

**Variable**	**Category**	**No Delay n (%)**	**Delay n (%)**	**χ^2**	**p-value**
**Age (years)**	<20	24 (82.8)	5 (17.2)	3.75	0.249
	20–29	160 (74.4)	55 (25.6)		
	30–39	139 (73.2)	51 (26.8)		
	>40	24 (64.9)	13 (35.1)		
**Urbanization**	Rural	89 (61.4)	55 (37.9)	**22.17**	**0.001**
	Semi-urban	78 (72.2)	30 (27.8)		
	Urban	181 (82.6)	39 (17.8)		
**Education**	Primary	56 (63.4)	33 (36.6)	14.00	**0.030**
	Secondary	185 (75.5)	60 (24.5)		
	Tertiary	106 (77.4)	31 (22.6)		
**Occupation**	Govt	53 (84.1)	10 (15.9)	**22.94**	**0.001**
	Housewife	62 (60.8)	40 (39.2)		
	Private	15 (60.0)	10 (40.0)		
	Self-emp	165 (77.8)	46 (21.7)		
	Student	53 (75.7)	18 (25.7)		
**Income (FCFA)**	<25k	137 (72.1)	52 (27.9)	12.91	**0.012**
	25–50k	92 (69.2)	41 (30.8)		
	51–100k	65 (76.5)	20 (23.5)		
	>100k	54 (84.4)	11 (15.6)		

Supplementary Table S2: Univariable analysis of factors associated with the third delay (delay in receiving adequate care at the health facility)

Univariable chi-square associations between sociodemographic variables and the third delay (N=472). Variables with $p < 0.05$ were considered for multivariable modelling.

**Variable**	**Category**	**No Delay n (%)**	**Delay n (%)**	**χ^2**	**p-value**
**Age**	<20	26 (89.7)	3 (10.3)	**11.18**	**0.011**
	20–29	151 (70.2)	64 (29.8)		
	30–39	**119 (62.6)**	**71 (37.4)**		
	>40	21 (56.8)	16 (43.2)		
**Urbanization**	Rural	**100 (69.0)**	44 (30.3)	6.31	**0.043**
	Semi-urban	**62 (57.4)**	**46 (42.6)**		
	Urban	155 (70.8)	64 (29.2)		
**Marital**	Married	**226 (71.7)**	88 (27.9)	**12.30**	**0.001**
	Single	89 (59.7)	61 (40.3)		
	Widowed	2 (28.6)	5 (71.4)		
**Income**	<25k	142 (74.7)	47 (24.7)	11.30	**0.005**
	25–50k	87 (65.4)	46 (34.6)		
	51–100k	**46 (54.1)**	**39 (45.9)**		
	>100k	42 (65.6)	22 (34.4)		
